# Predictors of Bovine TB Risk Behaviour amongst Meat Handlers in Nigeria: A Cross-Sectional Study Guided by the Health Belief Model

**DOI:** 10.1371/journal.pone.0056091

**Published:** 2013-02-11

**Authors:** Dupe Hambolu, Jenny Freeman, Henock B. Taddese

**Affiliations:** 1 Federal Department of Livestock and Pest Control Services, Federal Ministry of Agriculture and Water Resources, Abuja, Nigeria; 2 School of Health and Related Research (ScHARR), University of Sheffield, Sheffield, United Kingdom; San Francisco General Hospital, University of California San Francisco, United States of America

## Abstract

**Background:**

Bovine Tuberculosis (bTB) is still a serious public health threat in developing countries. The aim of this study is to determine the social and cognitive factors predicting one of the risk behaviours amongst meat handlers in Nigeria, namely, eating *Fuku Elegusi*. This is the practice of eating the visibly infected parts of the lung in-order to convince customers to buy meat. The study is guided by the health belief model (HBM).

**Methods:**

This is a cross-sectional study of 349 randomly selected meat handlers in Oko-Oba Abattoir, in Lagos State. Descriptive statistics and multiple logistic regression analysis were employed to determine perceptions and prevalence of risk behaviours and to identify predictors of eating *Fuku Elegusi*.

**Results:**

Just over a quarter (28.1%) of the study participants knew that eating *Fuku Elegusi* could be a source of bTB in humans. The prevalence of eating *Fuku Elegusi* was found to be 22%. Across all knowledge indicators related to bTB, those who don't eat *Fuku Elegusi* exhibited better knowledge. Strong predictors of eating *Fuku Elegusi* were: being male (OR: 2.39, 95% CI: 1.10 to 5.19; p = 0.03), not knowing that eating *Fuku Elegusi* exposes to bTB (OR: 3.72, 95% CI: 1.69 to 8.22; p = 0.001), and the perception that one cannot sell meat without tasting it (perceived barrier) (OR: 1.35, 95% CI: 1.13 to 1.60; p = 0.001). Lower risk of eating Fuku Elegusi was predicted by perceived susceptibility to bTB due to another risk behaviour, namely, not washing hands after handling meat (OR: 0.78, 95% CI: 0.64 to 0.96; p-value = 0.021). Television and radio were the most acceptable media for TB prevention messages (78.5% and 75.6% respectively).

**Conclusion:**

Meat handlers in developing countries bear high risk to bTB owing to prevailing social and cognition determinants. Findings were largely consistent with the propositions of HBM.

## Introduction

Tuberculosis (TB) continues to be a global priority disease. In 2010, an estimated 8.5–9.2 million TB cases were reported globally along with 1.2–1.5 million deaths (including deaths from TB among people living with HIV) [1]. Asia and Africa are disproportionately affected with 57% and 26% of the disease burden, respectively [1]. Global TB infection rates have seen an upsurge of up to 40% in the last three decades, which is attributable to the HIV/AIDS pandemic. The dual HIV-TB co-infection predominantly affects Sub Saharan Africa, which accounts for 82% of the World's HIV related TB [2].

Tuberculosis in human beings is mainly caused by *Mycobacterium tuberculosis (M. tb)*. However, bovine TB (bTB), the zoonotic form of TB caused by *M. bovis*, with cattle as the primary host, is a lingering concern in much of the developing world. In the developed world, eradication measures such as test-and-slaughter strategy and compulsory pasteurization of milk have led to effective control of the problem [3], [4], [5]. In developing countries, bTB is still an important zoonotic disease transmissible through inhalation of aerosols, which gives rise to pulmonary TB and through ingestion of contaminated milk and meat, which mostly causes extra pulmonary TB with lesions in lymph nodes, bones and joints, genitourinary system, and the meninges. It is also transmitted through a less common route of traumatic inoculation into the skin by those handling contaminated meat. There are only rare anecdotal records of human to human transmission in the case of *M. bovis*. The disease caused by *M. bovis* is clinically indistinguishable from that caused by *M. tb* [3], [6].

Although there is lack of data regarding the spread of bTB amongst human population in the developing world, global estimates attribute around 2.1% of pulmonary TB and 9.4% of extra-pulmonary TB cases to *M. bovis* [5], [7]. From review of the few studies conducted in Africa, it is estimated that M. bovis is attributable to about 5–7% of all human TB cases in the region [5]. These are still seen to be underestimations of the problem due to lack of reliable data [3], [4], [5], [8]. In developing countries, the risk of infection by bTB is exacerbated due to: close contact between cattle and human beings as large proportions of the populations in these settings depend on agriculture for their livelihoods, practices of drinking raw milk and eating uncooked or partially cooked meat, and social and economic challenges that constrain use of effective control measures. The high HIV infection rates and the associated increase in susceptibility to TB and other infections is also a critical factor in these settings. TB represents a complex, long standing problem in these settings as it is embedded in deep social, economic and cultural determinants: high stigma attached to the disease, low awareness and low health literacy, poverty, poor sanitation, and crowded dwellings [3], [6], [9], [10].

While there is shortage of data regarding the contribution of bTB to the overall human TB prevalence in Nigeria, molecular analysis of mycobacterial strains isolated from both pulmonary and extra pulmonary TB cases have indicated that up to 14% of them belong to *M. bovis* [9]. The fact that *M. bovis* has been isolated from various animal products such as fresh and sour milk, from lesions in the lung and lymph nodes at slaughterhouses, as well as from sputum and biopsy samples of humans, indicates that the disease spreads through both direct and indirect modes of transmission (through inhalation and ingestion of milk and uncooked meat) [9], [11], [12], [13]. These studies have further shown that herdsmen, abattoir workers, and other handlers of livestock and livestock products are at a high risk of infection. [9]. A recent cross-sectional study that screened sputum samples from livestock traders in Nigeria found high prevalence of TB (10%) amongst this group and further revealed that 2 out of 7 of the identified TB cases were caused by M. bovis strains [14]. The study further emphasised that the susceptibility of this group to TB emanated from their occupational exposure as well as the general poor, overcrowded living conditions.

The World Health Organisation (WHO) and the Food and Agriculture Organisation (FAO) have long emphasised the need to improve the collection of scientific data on bTB to enhance understanding of effects on, and patterns of transmission in, affected populations [3], [15]. This study set out to explore the cognitive and social predictors of a high risk, customary behaviour amongst meat handlers in Nigeria, namely, eating *Fuku Elegusi*, that is, eating the visibly infected parts of the lungs in order to reassure customers that the meat on display is safe to eat. Given the high prevalence of both HIV and TB in the country, and the particular susceptibility of the population to bTB, it is important to understand behavioural patterns that increase susceptibility to the disease along with exploring entry points for effective action for prevention and control of the disease.

### The Theoretical Framework of the Study

The health belief model (HBM) constructs [16] were used to operationalise the theoretical proposition of the study through guiding the formulation of the specific objectives, development of the survey questionnaires and analysis of the findings. HBM posits that health related decisions depend on the combined effects of: one's perceptions of susceptibility to a given condition and severity of the condition, which together make up ‘perceived threat’; perceptions of barriers or costs to adopting preventative or curative action; perceptions of the benefits of engaging in specified health actions; and perceptions on available alternatives for adoption of preventive or curative behaviour, that is, cues to action. Behavioural change is also modified by factors such as demographic variables, knowledge, and societal influences. Self efficacy, an individual's perceptions of his/her self competence to be able to successfully execute actions required to bring about desirable health outcomes, was later added to the model [17].

Therefore, according to HBM, a meat trader in an abattoir is likely to uphold health-related precautionary measures, such as avoiding eating raw meat or not selling contaminated meat, if he/she considers bTB to be a serious health threat and believes himself/herself to be susceptible to bTB. In other words, a meat trader is less likely to eat the visibly infected parts of the lung (Fuku Elegusi) when they feel they are at a heightened risk of bTB owing to their general work conditions and their day to day activities and habits, such as, working in the abattoir, processing raw meat with inadequate protective wear and not washing their hands after processing meat. A meat trader is also likely to heed health related messages if he/she believes that the benefits of the new precautionary measures taken to avert bTB outweigh the costs, and if societal factors have a potentiating rather than a hindering effect. The person will also need to feel that they are capable of undertaking the required actions to avoid risky behaviours (self-efficacy). The cues to action construct is the least systematically studied or understood of all constructs [16]; our adoption of this concept in this study is limited to identifying the most preferred means of communication amongst respondents for bTB prevention and control measures.

HBM has been applied across various health promotion programmes such as vaccination against infectious diseases, breast self examination for early detection of cancer, smoking cessations, dieting and seat belt use [18], [19], [20]. This study is the first of its kind regarding the application of the model to study health behaviour related to zoonotic diseases in developing countries.

## Materials and Methods

The study was conducted in Oko-Oba Abattoir facility, in Agege County. This abattoir, customarily referred to as Agege Abattoir, is found in the suburb of Lagos State, in the Western part of Nigeria [21], [22]. It is the largest abattoir in the State and receives cattle from Northern Nigeria as well as wider geographical areas, such as, some neighbouring countries including Niger, Chad, Burkina Faso, Mali and Cameroon [21], [23]. Over one thousand cattle are slaughtered per day at the abattoir [21]. Although it is a state property, the abattoir is run by private contractors who collect revenue from the butchers per head of cattle [21].

Questionnaires were administered through face-to-face interviews with 349 meat handlers who were 18 years or above, working in Oko-Oba Abattoir. Out of 1,512 meat handlers who were on the abattoir's registry, a total of 385 meat traders (25%) were selected using the systematic random sampling method [24]. However, only 349 respondents were willing to participate in the study. Data was collected using a structured questionnaire, over 2 weeks, from the 24^th^ of June to the 8^th^ of July, 2011. The questionnaire was divided into four sections. The first section included questions on participants' age, gender, marital status, level of education, monthly income, religion, tribe and years of business experience. The second section comprised 6 questions eliciting knowledge on bTB, with response options of ‘yes’, ‘no’ or ‘I don't know’. The third section had 5 items inquiring about risk taking behaviour, including whether participants ate *‘Fuku Elegusi’*, with response options of either ‘yes’ or ‘no’. The fourth and final section consists of questions relating to each of the health belief model constructs: perceived susceptibility (5 questions), perceived severity (5 questions), perceived barriers (5 questions), self efficacy (3 questions) and cues to action (7 questions). For the items in the health belief model constructs, participants were asked to indicate their extent of agreement to statements eliciting their views on a 5-point likert scale: 5) Strongly Agree 4) Agree 3) Neither agree nor disagree 2) Disagree 1) Strongly disagree.

### Data Collection and Data Analysis

The main study outcome was whether respondents did or did not eat *Fuku Elegusi*. Those who ate *Fuku Elegusi* were classified as high risk and those who did not were classified as low risk. The independent variables were related to: demographic variables, knowledge indicators related to TB and bTB, other risky behaviours related to bTB, participants' perceived susceptibility to bTB, perceived severity, perceived barriers, self-efficacy and cues to action.

Initially data were tabulated, both for all participants and by risk group (eats/does not eat *Fuku Elegusi*). As all the variables were categorical, the values in each category are presented together with their corresponding percentages. Univariate analyses (using chi-squared statistic with Fisher's exact test when needed) were conducted to identify potential candidate variables for the main logistic regression model. . For ordered variables or those measured on an ordinal likert scale, the Mann Whitney U test was used.

Following this stage, multiple logistic regression analysis was conducted to examine the effects of the independent variables on risk. Candidate variables were included in the model if their p-value was less than 0.10 in the univariate analysis. Backwards stepwise regression was used with the least significant variable removed at each stage until the model contained only those factors that were significant at the 5% level. Statistical analyses were conducted using the Statistical Package for the Social Science (SPSS) version 19.0 and a p-value of less than 0.05 was used to define statistical significance.

Ethical approval was obtained from the School of Health and Related Research (ScHARR), University of Sheffield, UK, as well as from the Ministry of Agriculture, Lagos State, Nigeria. Information sheet were read out to the participants and written informed consents were obtained through signature or thumb printing on consent forms.

## Results

In total, 349 people responded to the questionnaires. Of these, 75 (21.5%) respondents reported eating *Fuku Elegusi*, placing them at higher risk of bTB (Table 1). In this study, those eating *Fuku Elegusi* are referred to as ‘the high risk group’ denoting that they are engaging in a high risk behaviour, whereas those who confirmed that they do not eat Fuku Elegusi are in turn referred to as ‘the low risk group’. For the demographics there was little evidence that the groups differed except for sex, where there was a tendency for the *Fuku Elegusi* (high risk behaviour) group to have a higher percentage of men (88.0% men in the *Fuku Elegusi* group vs 75.5% men in the *non-Fuku Elegusi* group, p = 0.031). Although not significant there was a suggestion that length of working in the industry was higher for the high risk group (p = 0.06) (Table 2).

**Table 1:** Prevalence of high risk behaviours (n = 349).

	N (%; 95% CI)
Do not wear protective clothing when handling raw meat	310 (88.8; CI: 85.1 to 91.7 )
Sell meat even if has signs of contamination	98 (28.1; CI: 23.6 to 33.0)
Eat Fuku Elegusi meat before selling	75 (21.5; CI: 17.5 to 26.1)
Eat raw meat before selling	50 (14.3; CI: 11.0 to 18.4)
Do not wash hands after handling raw meat	49 (14.0; CI: 10.8 to 18.1)

**Table 2:** Demographics, total and by whether they eat Fuku Elegusi (n = 349 unless otherwise stated).

			By risk category		
		Total n (%)	Does not eat Fuku Elegusi (n = 274) %	Eats Fuku Elegusi (n = 75) %	P-value
Age (n = 348)	21–30	62 (17.8)	18.3	16.0	0.84
	31–40	121 (34.8)	34.4	36.0	
	41–50	90 (25.9)	26.0	25.3	
	51–60	64 (18.4)	17.2	22.7	
	62–70	11 (3.2)	4.0	-	
Gender	Male	273 (78.2)	75.5	88.0	0.031
	Female	76 (21.8)	24.5	12.0	
Tribe	Yoruba	271 (77.7)	78.1	76.0	0.75*
	Hausa	65 (18.6)	17.9	21.3	
	Ibo	13 (3.7)	4.0	2.7	
Education	None	196 (56.2)	54.4	62.7	0.43
	Primary	100 (28.7)	30.7	21.3	
	Secondary	39 (11.2)	10.6	13.3	
	Tertiary	14 (4.0)	4.4	2.7	
Religion (n = 348)	Muslim	255 (73.3)	73.4	73.0	0.994
	Christian	88 (25.3)	25.2	25.7	
	Other	5 (1.4)	1.5	1.4	
Marital status	Single	51 (14.6)	14.6	14.7	0.88*
	Married/co-habiting	277 (79.4)	79.2	80.0	
	Separated/divorced	18 (5.2)	5.5	4.0	
	Widowed	3 (0.9)	0.7	1.3	
Length of time in business	1–10 years	48 (13.8)	15.0	9.3	0.08
	11–20 years	67 (19.2)	19.7	17.3	
	21–30 years	130 (37.2)	37.2	37.3	
	31–40 years	86 (24.6)	24.1	26.7	
	41–50 years	18 (5.2)	4.0	9.3	

* Fisher's exact test.

Over 80% had heard of bTB but only 31% knew that it could spread from animals to humans (bTB) and less than 20% were aware that even healthy looking meat could be contaminated (18.9%) (Table 3). Just over a third of respondents (34.1%) knew that consumption of infected meat could be a source of bTB in humans and just over a quarter (28.1%) knew that consumption of *Fuku Elegusi* could be a source of bTB in humans. Comparison between the two risk groups (those who eat *Fuku Elegusi* and those who don't) revealed that on almost all questions the two groups differed, with the low risk group exhibiting better knowledge on all accounts except for whether bTB could be transmitted from animals to humans (p = 0.12) and modes of transmission (p = 0.08) (Table 3). For this latter variable, although it was not statistically significant, the low risk group still demonstrated better knowledge about bTB compared to the high risk group.

**Table 3:** Knowledge of BTB, by risk category (n = 349).

			By risk category		
		Total n (%)	Does not eat Fuku Elegusi (n = 274) %	Eats Fuku Elegusi (n = 75) %	P-value
Have you heard of TB?					
	No	55 (15.8)	12.8	26.7	0.007
	Yes	294 (84.2)	87.2	73.3	
Can TB be spread from animals to humans?					
	No	116 (33.2)	33.6	32.0	0.12
	Yes	107 (30.7)	32.8	22.7	
	Don't know	126 (36.1)	33.6	45.3	
How is TB spread from animals to humans?					
	Aerosol (air-bourne)	9 (2.6)	2.9	1.3	0.08*
	Contaminated milk	15 (4.3)	5.1	1.3	
	Under-cooked contaminated meat	142 (40.7)	42.0	36.0	
	All of the above	29 (8.3)	9.5	4.0	
	Don't know	154 (44.1)	40.5	57.3	
Can healthy looking meat contain TB?					
	No	138 (39.5)	40.5	36.0	0.001
	Yes	66 (18.9)	22.3	6.7	
	Don't know	145 (41.5)	37.2	57.3	
Is consumption of contaminated meat a source of BTB infection in humans?					
	No	94 (26.9)	24.5	36.0	0.002
	Yes	119 (34.1)	38.7	17.3	
	Don't know	136 (39.0)	36.9	46.7	
Is consumption of Fuku Elegusi meat a source of BTB infection in humans?					
	No	106 (30.4)	29.9	32.0	<0.001
	Yes	98 (28.1)	32.8	10.7	
	Don't know	145 (41.5)	37.2	57.3	

* Fisher's exact test.

The evidence for the respondents' perceived susceptibility was less clear-cut than for their knowledge with only the question regarding hand washing showing a significant difference (Table 4). In this case, the difference was in the expected direction, with the low risk group more likely to agree that not washing their hands after handling carcasses puts them at greater risk (p = 0.004). A related question about being at greater risk when using bare hands showed an effect in the same direction but was not statistically significant (p = 0.07).

**Table 4:** Perceived susceptibility (values are %).

	Strongly disagree	Disagree	Neither agree nor disagree	Agree	Strongly agree	P-value
I have an increased chance of contracting BTB because of my work:						
Low risk (n = 274	13.1	20.1	23.7	21.5	21.5	0.17
High risk (n = 75)	16.0	22.7	25.3	22.7	13.3	
I am at increased risk of contracting BTB when I use bare hands						
Low risk (n = 274)	13.1	20.1	23.7	16.8	25.9	0.07
High risk (n = 75)	17.3	22.7	25.3	22.7	12.0	
I am at increased risk of contracting BTB when I eat on the slaughter slab						
Low risk (n = 274)	22.3	22.6	26.3	14.2	14.6	0.21
High risk (n = 75)	24.0	22.7	37.3	9.3	6.7	
I am at increased risk of contracting BTB when I don't wash my hands after handling carcasses						
Low risk (n = 274	8.0	13.9	17.2	26.3	34.7	0.004
High risk (n = 75)	12.0	17.3	25.3	29.3	16.0	
I am at increased risk of contracting BTB when I eat raw meat						
Low risk (n = 274)	7.3	8.4	15.3	26.6	42.3	0.12
High risk (n = 75)	14.7	5.3	17.3	29.6	33.3	

In general, the low risk group were more likely to agree that TB and its consequences were serious (Table 5). They were more scared of TB (p = 0.03), and more likely to agree that it would keep them in bed for an extended period of time (p = 0.03). Interestingly, they were also more likely to agree that it was treatable (p = 0.008). There was no difference between the two risk groups in terms of whether or not they think that contracting TB would prevent them from coming to work, or whether it could cause death.

**Table 5:** Perceived severity (values are %).

	Strongly disagree	Disagree	Neither agree nor disagree	Agree	Strongly agree	P-value
Contracting BTB will prevent me coming to work						
Low risk (n = 274)	4.4	8.0	14.6	29.6	43.4	0.14
High risk (n = 75)	5.3	16.0	14.7	26.7	37.3	
Contracting BTB will keep me in bed for an extended period of time						
Low risk (n = 274)	5.8	9.5	22.3	25.9	36.5	0.03
High risk (n = 75)	5.3	17.3	26.7	26.7	24.0	
Contracting BTB scares me						
Low risk (n = 274)	3.3	6.2	12.0	32.5	46.0	0.03
High risk (n = 75)	6.7	12.0	14.7	30.7	36.0	
BTB can cause death						
Low risk (n = 274)	6.2	9.9	13.1	31.0	39.8	0.32
High risk (n = 75)	6.5	8.0	14.7	41.3	29.3	
TB is treatable						
Low risk (n = 274)	13.9	13.5	25.9	26.6	20.1	0.008
High risk (n = 75)	16.0	18.7	36.0	24.0	5.3	

There were no differences in the perceived barriers to prevention except for whether they needed to taste the meat before selling it (Table 6). In this case, the low risk group were less likely to agree that they needed to taste the meat before selling it (p<0.001). Two of the three items making up the self-efficacy construct were not statistically significant (Table 7). The two groups only differed in terms of whether they were able to differentiate infected carcasses, with the low risk group more likely to agree that they were able to do that (p = 0.02).

**Table 6:** Perceived barriers to prevention (values are %).

	Strongly disagree	Disagree	Neither agree nor disagree	Agree	Strongly agree	P-value
I need to taste meat before selling to show that it is safe						
Low risk (n = 274)	40.5	29.2	7.3	5.8	17.2	<0.001
High risk (n = 75)	18.7	28.0	12.0	17.3	24.0	
I can't wear protective clothing because they are not conducive for work						
Low risk (n = 274)	26.3	27.0	13.9	15.0	17.9	0.25
High risk (n = 75)	16.0	36.0	9.3	18.7	20.0	
I can't wear protective clothing because they are expensive						
Low risk (n = 274)	29.2	33.9	19.7	10.9	6.2	0.19
High risk (n = 75)	26.7	29.3	17.3	13.3	13.3	
I don't wear protective clothing because my colleagues do not						
Low risk (n = 274)	33.9	33.2	17.5	10.6	4.7	0.92
High risk (n = 75)	32.0	41.3	9.3	9.3	8.0	
Confiscating my meat will put me out of business as there is inadequate compensation						
Low risk (n = 274)	36.1	23.0	18.2	10.9	11.7	0.09
High risk (n = 75)	45.3	24.0	16.0	4.0	10.7	

**Table 7:** Self- efficacy (values are %).

	Strongly disagree	Disagree	Neither agree nor disagree	Agree	Strongly agree	P-value
I can buy protective wear						
Low risk (n = 274)	8.0	10.9	9.1	33.9	38.0	0.56
High risk (n = 75)	4.0	10.7	17.3	36.0	32.0	
I can wear protective wear even if my colleagues are not						
Low risk (n = 274)	2.9	10.2	8.4	31.4	47.1	0.12
High risk (n = 75)	2.7	14.7	13.3	30.7	38.7	
I am able to tell if carcasses are infected with TB						
Low risk (n = 274)	11.7	17.9	22.6	30.7	17.2	0.02
High risk (n = 75)	18.7	17.3	29.3	29.3	5.3	

**Figure 1:**
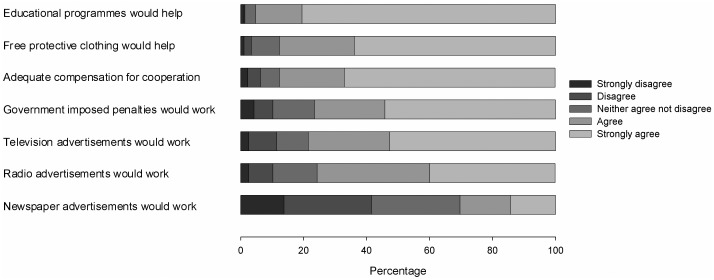
Cues to action, n = 349. The most popular interventions for facilitating the adoption of protective behaviours and practices were: educational programmes, supply of free protective clothing, adequate compensation for cooperating with test and slaughter campaigns, government imposed penalties, and television and radio advertisements for dissemination of positive health seeking behaviour re-enforcing messages. More than 75% of respondents either strongly agreed or agreed to these suggested interventions. In contrast, just about 30% agreed or strongly agreed to newspaper adverts.

Finally, when looking at cues to action, the overwhelming majority of respondents agreed or strongly agreed that educational programmes and free provision of protective clothing would help (95.2% and 87.7% respectively) (Figure 1). In addition, 87.6% of respondents felt that they would need adequate compensation in order to comply with meat inspections and 76% felt that government imposed penalties for those who do not practise safe measures would work. Both television and radio advertisements on TB control were popular with respondents (78.5% and 75.6% respectively) whereas only a minority were in favour of newspaper advertisements (30.3%).

When the impact of the many potential predictor variables outlined above was investigated using multiple logistic regression, after adjustment for sex, three variables remained important in determining the risk of eating *Fuku Elegusi (* *Table 8* *)*. The Hosmer Lemeshow chi-squared statistic for the final model was 8.29 of 8 degrees of freedom (p = 0.46), indicating that there is no evidence of a lack of fit – the model fitted the data well. All the factors increased the risk of eating *Fuku Elegusi*, with the exception of the variable relating to washing hands after handling raw meat. Regarding the sex of participants, the odds of eating *Fuku Elegusi* for males was 2.39 times greater than the odds for females (95% CI: 1.10 to 5.19; p = 0.03). Those who did not think that eating *Fuku Elegusi* was a risk factor for bTB or who were unsure were more likely to eat *Fuku Elegusi* (OR: 3.72, 95% CI: 1.69 to 8.22; p = 0.001) as were those who felt that there was a need to taste the meat before selling it (OR: 1.35, 95% CI: 1.13 to 1.60; p = 0.001). The odds of eating *Fuku Elegusi* were lower only for those who agreed that the risk of bTB was higher if you did not wash your hands after handling raw meat (OR: 0.78, 95% CI: 0.64 to 0.96; p-value = 0.021).

**Table 8:** Results of logistic regression analysis (n = 349).

Risk factor		Odds ratio (95% CI)	P-value
Gender:	Male (compared to female)	2.37 (1.10 to 5.14)	0.028
Eating Fuku Elegusi is a risk factor for BTB	No/don't know (compared to yes)	3.72 (1.69 to 8.22)	0.001
Need to taste meat before selling it	No/don't know (compared to yes)	1.35 (1.13 to 1.60)	0.001
At risk if don't wash hands after handling raw meat	No/don't know (compared to yes)	0.78 (0.64 to 0.96)	0.021

## Discussion

This study of the predictors of a high risk behaviour amongst meat traders in Nigeria, that is, eating *Fuku Elegusi* (*M. bovis* infected parts of the lungs), was guided by the health belief model. This has enabled identification and testing of key social and cognitive factors in determining this customary, high risk practice. The study has established the high prevalence of risky behaviours for bTB, including the primary outcome of interest in this study, that is, Fuku Elegusi. These include: eating *Fuku Elegusi* (22%), not wearing gloves while processing meat (89%), eating raw meat (14%), selling meat even when visibly contaminated (28%) and not washing hands after processing raw meat (14%). Although 84% claimed they were aware of tuberculosis, the level of knowledge related to bovine tuberculosis and its means of transmission is very disheartening for people drawn from a high risk occupation; one would expect them to be prioritized with messages pertaining to BTB [4], [5].

This is consistent with findings from the few studies that assessed knowledge and behaviour amongst high risk groups in similar settings in other African countries. Swai et al assessed the knowledge and practice of animal health workers and livestock keepers in Tanzania. They concluded that, ‘patchy awareness and lack of knowledge of zoonoses combined with food consumption habits and poor animal husbandry are likely to expose respondents to an increased risk of contracting zoonoses’ [25]. Similarly, Mfinanga et al investigated the level of knowledge and prevailing practices in rural Tanzania and found that about 40% of respondents practice habits deemed to be ‘high risk’ for exposure to bTB, while 75% exhibited poor knowledge of TB [26]. Amenu et al also documented the lack of accurate knowledge on transmission of zoonoses and the prevalence of risky behaviour, such as consumption of raw animal products and unsafe slaughtering practices in a rural district in Ethiopia [27].

Out of the demographic factors, sex was found to be important as a predictor of the high risk behaviour, that is, consumption of *Fuku Elegusi*. Social science theorists have provided explanations of observed predominance of risk taking behaviour amongst males as inherently linked to the social construction of masculinity [28], [29]. However, specific contexts would need to be studied in order to develop an in-depth understanding of the complex mechanisms in which the social, cultural and institutional circumstances across a range of settings give rise to such differences in health related behaviour [28], [29]. Complementing this study of relationships between variables with an in-depth exploration of underlying social and cultural factors (a qualitative study), as part of a mixed methods approach [30], would help to improve understanding of how risk-taking is mediated in this male dominated occupation.

The low risk group was found to exhibit better knowledge. This is in line with theories of the health belief model as well as other health behaviour models, where the models recognise the critical importance of awareness or conscious-raising of populations for promotion of positive health reinforcing behaviour [18], [19]. The investigation of cues to action has in turn revealed the most likely popular medium for delivery of pertinent information, education and communication (IEC) materials; audio-visual means such as TV and radio were favoured to print media such as newspapers for propagating health education messages. This information can be used to guide subsequent behavioural change communication (BCC) work in the area.

The findings pertaining to the health belief model constructs were in the expected direction with different items under each of the constructs showing that the low risk group were more likely to perceive the risk and severity of bTB. The low risk group were more likely to confirm capacity in terms of having skills that could help in reinforcing desirable health behaviour (self-efficacy) and less likely to reveal the presence of barriers between their current behaviour and the desirable actions. Items from perceived susceptibility and perceived barriers, namely the perception that not washing hands after handling raw meat exposes to bTB and the perception that one cannot sell meat without tasting, were the strongest predictors of risky behaviour. This is consistent with findings from Janz and Beckers review of 29 studies that tested the health belief model between 1974 and 1984. According to their analysis, the perceived barriers were the most important predictors of behaviour while perceived susceptibility was the most important amongst predictors of preventative behaviour (where the study subjects were people not affected by a disease or condition and the assessed behaviour pertains to preventative actions) in contrast to ‘sick-role’ behaviours (where the study subjects were people affected by a disease condition and the assessed behaviour was related to habits that exacerbated their conditions or supported treatment plans) [16], [20].

The findings of this study are generalisable to meat handlers in Nigeria. The chosen abattoir for the study, Oko-Oba (Agege) Abattoir is typical of other abattoirs in the country with respect to the conditions of the abattoir and how meat handlers are regulated in the abattoir. Accordingly, findings of low knowledge levels and high prevalence of risk behaviours portend negatively to trends amongst meat traders in Nigeria. The analysis of cognitive and socio-demographic determinants of the selected risky behaviour (*Fuku Elegusi*) generates critical insights into such socio-culturally embedded practices amongst such groups. In addition, the study has identified acceptable media for health education. These findings can in turn inform policy and action aimed at addressing the susceptibility of this risk group, as well as providing testable propositions for further study in the area.

One of the limitations of this study is linked to the adopted cross-sectional study design, whereby claims about causal relationships between the dependent and independent variables cannot be verified. Reviews have demonstrated a clear superiority of longitudinal designs in studies of belief-behaviour relationships [31]. The administration of the questionnaires through face to face interviews may increase likelihood of respondents' inclination to give socially acceptable answers. More comprehensive and in-depth exploration of the factors would have been generated by also employing qualitative methods, such as in-depth interviews, within a mixed methods study design. Despite these limitations, there was a high response rate (90.6%), making the results likely to be representative of the population.

## Conclusion

This study adds to existing evidence as to the critical lack of accurate knowledge and the prevalence of high risk behaviour amongst meat traders in Africa. The study further highlights the preferred media for IEC/BCC work aimed at bringing about desirable behaviour change and sheds light on the acceptability of institutional and regulatory action to facilitate desirable behaviour in the setting. This information can serve as critical input for health programmes aiming to tackle the highlighted gaps. By testing the health belief model constructs, the study further presents the most important cognitive determinants, which in-turn would be valuable inputs in designing behavioural change strategies. In addition, use of the widely applied theoretical framework has made the findings readily testable for studies conducted in similar settings. Further studies that also employ more interpretive approaches are recommended to help understand the complex pathways in which the multiple social and cognitive factors influence behaviour related to BTB and other zoonotic diseases in such settings.
